# 3D CNN classification model for accurate diagnosis of coronavirus disease 2019 using computed tomography images

**DOI:** 10.1117/1.JMI.8.S1.017502

**Published:** 2021-07-23

**Authors:** Yifan Li, Xuan Pei, Yandong Guo

**Affiliations:** a Beijing University of Posts and Telecommunications, Beijing, China; b OPPO Research Institute, Shenzhen, China

**Keywords:** COVID-19, computer tomography, deep learning, classification network, radiography

## Abstract

**Purpose:** The coronavirus disease (COVID-19) has been spreading rapidly around the world. As of August 25, 2020, 23.719 million people have been infected in many countries. The cumulative death toll exceeds 812,000. Early detection of COVID-19 is essential to provide patients with appropriate medical care and protecting uninfected people.

**Approach:** Leveraging a large computed tomography (CT) database from 1112 patients provided by China Consortium of Chest CT Image Investigation (CC-CCII), we investigated multiple solutions in detecting COVID-19 and distinguished it from other common pneumonia (CP) and normal controls. We also compared the performance of different models for complete and segmented CT slices. In particular, we studied the effects of CT-superimposition depths into volumes on the performance of our models.

**Results:** The results show that the optimal model can identify the COVID-19 slices with 99.76% accuracy (99.96% recall, 99.35% precision, and 99.65% F1-score). The overall performance for three-way classification obtained 99.24% accuracy and a macroaverage area under the receiver operating characteristic curve (macro-AUROC) of 0.9998. To the best of our knowledge, our method achieves the highest accuracy and recall with the largest public available COVID-19 CT dataset.

**Conclusions:** Our model can help radiologists and physicians perform rapid diagnosis, especially when the healthcare system is overloaded.

## 1 Introduction

The outbreak of the 2019 novel coronavirus (SARS-CoV-2) began in early December 2019.^1^^,^^2^ The infection has an average incubation period of 5.2 days and can cause fever, cough, and other flu-like symptoms. It can affect multiple tissues and organ systems, and diseases caused by viruses are collectively referred to as coronavirus disease 2019 (COVID-19). Many infected patients develop pneumonia and rapidly, severe acute respiratory failure, with very poor prognosis and high mortality.^3^^,^^4^ Person-to-person transmission was reported as one of the possible routes.^5^^–^^8^ Compared with the prior Severe Acute Respiratory Syndrome (SARS) and Middle East Respiratory Syndrome, although COVID-19 has a relatively lower fatality rate and spread to more places and caused more deaths.^9^^,^^10^ The COVID-19 outbreak was declared a pandemic by the World Health Organization. Therefore, it is necessary to build an accurate diagnostic solution for early intervention and close monitoring of COVID-19, helping to slow the spread of the virus and contain the disease.

In clinics, a positive molecular polymerase chain reaction (PCR) test is the gold standard for definitively diagnosing COVID-19.^11^ However, the high false-negative rate^5^ and unavailability of PCR assay in the early stage of an outbreak may delay the identification of potential patients.^12^ Chest computed tomography (CT) is an important tool for diagnosing lung diseases, including pneumonia. CT scan procedures have a faster turnaround time than molecular diagnostic tests performed in standard laboratories and can provide more detailed pathological information. For example, almost all COVID-19 patients have some typical radiographic features in chest CT, including ground-glass opacities (GGOs), multifocal patchy consolidation, and/or interstitial changes with a peripheral distribution.^13^ Therefore, chest CT has been recommended as a major tool for clinical diagnosis, especially in the hard-hit region such as Hubei province, China.^11^ Since seasonal influenza can also cause viral pneumonia, it is also crucial to distinguish COVID-19 from common influenza or other types of pneumonia such as viral and bacterial pneumonia. Considering the high demand for chest CT screening and the workload of radiologists, especially as an outbreak occurs, we designed a deep-learning method using CT images to classify COVID-19, common pneumonia (CP), and normal controls.

In recent years, the application of deep learning in many medical fields has made exciting progress,^14^^–^^20^ stimulating the development and innovation of new radiological diagnostic technology. With the outbreak of the epidemic, deep-learning methods were used in the diagnosis, prognosis, detection, and treatment of COVID-19. Ouyang et al.^12^ developed a dual-sampling attention network to diagnose COVID-19 from CP in chest CT automatically and calculated an accuracy of 87.5%. Zhang et al.^21^ developed an artificial intelligence (AI) system that could diagnose COVID-19 and provide accurate clinical prognosis.

However, the current experimental results are mainly based on datasets with small size or expensive segmentation labels. There is a lack of relatively independent end-to-end COVID-19 classification research on a large dataset. In addition, these studies rarely consider the effect of CT scan layers or the input size of the model on the performance. This paper studies the end-to-end classification effect of three-dimensional (3D) ResNet and (2+1)D ResNet models on the largest public CT scan dataset for COVID-19 classification so far. The effects of different inputs of the model on the experimental results were studied, too.

As a summary, the contributions of our work are threefold:

(1) We investigated several 3D CNN technologies, including basic block, bottleneck block, and (2+1)D convolution and reported the optimal solution for detecting COVID-19 from CT images.
(2) We used different depths to superimpose CT slices for preprocessing to obtain more information between CT slices. The superimposed volume was used as the input of the 3D classification network. The experimental results demonstrate that the depth of volume has a significant influence on the model effect.
(3) We conducted experiments with a large CT dataset provided by the China Consortium of Chest CT Image Investigation (CC-CCII),^21^ including complete CT slices and segmented CT slices. Experimental results demonstrate that our method can identify the COVID-19 slices with 99.76% accuracy, 99.96% recall, 99.35% precision, and 99.65% F1-sorce. The overall performance for three-way classification obtained 99.24% accuracy and a macroaverage area under the receiver operating characteristic curve (macro-AUROC) of 0.9998. To the best of our knowledge, this is the most accurate result with the largest public available dataset.

## 2 Materials and Methods

### 2.1 Data Set

**Fig. 1:**
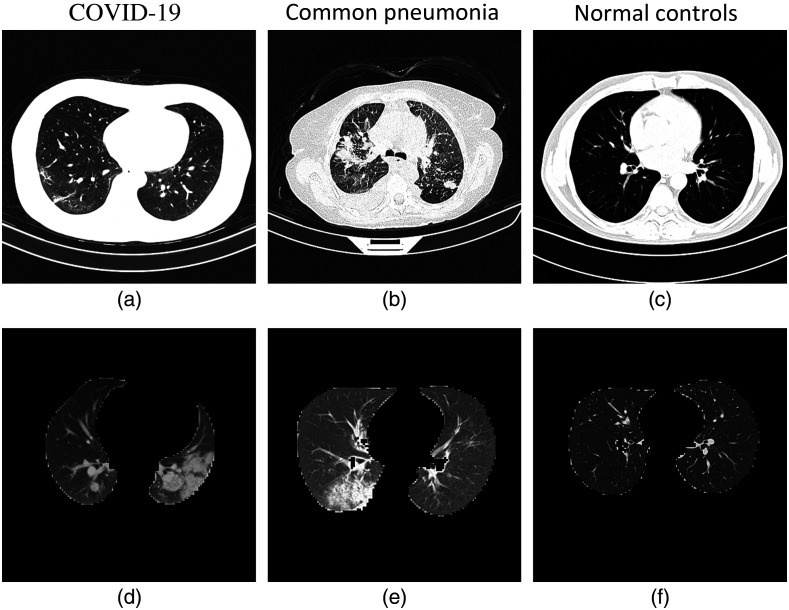
Typical transverse-section CT images: (a) complete CT images and (b) segmented CT images.

A large CT dataset from the CC-CCII was used^21^ in this paper; it consists of a total of 137,256 complete CT images from 691 patients and 42,861 segmented CT images from 421 patients (Fig. 1). Institutional Review Board (IRB)/Ethics Committee approvals were obtained in all of the institutions involved, and consent was obtained from all participants. The dataset of raw chest CT images and clinical metadata is available through the China National Center for Bioinformation at the website in Ref. 22. The original CT image dimensions in the CC-CCII dataset are 512*512.

A total of 110,420 complete CT images (80.4%) were employed to train and validate our model for discriminating COVID-19 from other CP and normal controls (Table 1). The remaining 26,836 CT images (19.6%) were used as the test set. In addition, the test set used CT slices selected from the individuals who were not included in the training and validation stages. Viral pneumonia, bacterial pneumonia, and mycoplasma pneumonia are included in the CP group, all of which are the most common causes of pneumonia in China. We also tried to use segmented CT images to train, validate, and test our model (Table 2).

**Table 1:** Complete CT dataset of characteristics in identifying COVID-19 from other CP and normal controls.

Complete CT	COVID-19			CP			Normal controls		
	Patients	Scans	Slices	Patients	Scans	Slices	Patients	Scans	Slices
Train	149	270	28,088	112	146	27,252	140	308	27,456
Valid	53	92	9364	45	52	9108	54	85	9152
Test	44	87	9236	44	44	8520	50	102	9080
Total	246	449	46,688	201	242	44,880	244	495	45,688

**Table 2:** Segmented CT dataset of characteristics in identifying COVID-19 from other CP and normal controls.

Segmented CT	COVID-19			CP			Normal controls		
	Patients	Scans	Slices	Patients	Scans	Slices	Patients	Scans	Slices
Train	79	98	11,648	2	3	222	175	175	14,144
Valid	26	26	3904	1	1	77	56	56	4736
Test	25	32	3456	1	1	66	56	56	4608
Total	130	156	19,008	4	5	365	287	287	23,488

### 2.2 Preprocess

CT slices were normalized to 512*512*3 for the height, width, and channel, respectively. To leverage the 3D volume of CT images to capture a wide range of spatial information both within the CT slices and between CT slices,^23^ n adjacent CT slices in the same CT scan were stacked vertically to form a volume, where n denotes the depth in the 3D volume. Depth can be regarded as the height of a CT scan from a 3D perspective or the number of slices after downsampling from one CT scan. We then transposed the volume from D×H×W×C (D denotes depth, H denotes height, W denotes width, and C denotes channel) to C×D×H×W, to derive a tensor. The diagnostic classifier took the tensor as input and used the classification network to generate the three-level probabilities of COVID-19, CP, and normal controls, predicting the volume’s label with the maximum probability after the softmax activation function.

### 2.3 Network Architecture

The detailed structure of the three-way classification network was shown in Table 3, based on the 3D ResNet-18 network.^26^ The network used multiple 3D basic blocks with residual connections that could continuously extract local and global contextual features and used a fully connected layer followed by the softmax activation function to calculate final predictions with the maximum probability for three types of diagnostic results.

**Table 3:** Network architectures. Each convolutional layer is followed by batch normalization^24^ and a ReLU activation function.^25^ Downsampling is performed in the first convolutional layer of each block with the stride of 2. F is the number of feature channels corresponding in Fig. 2, and N is the number of blocks in each layer.

Layer name	Architecture					
	18-layer		34-layer		50-layer	
	F	N	F	N	F	N
Conv1	7×7×7, 64, stride 1 (D), 2 (HW)					
Conv2	3×3×3 max pool, stride 2					
	64	2	64	3	64	3
Conv3	128	2	128	4	128	4
Conv4	256	2	256	6	256	6
Conv5	512	2	512	3	512	3
	Global average pool, fully connected, softmax layer					
Block	Basic		Basic		Bottleneck	

The cross-entropy was employed as the loss function between the final predictions and ground truth labels to train the 3D classification network. The Adam optimizer with an initial learning rate at 0.001 was used in the training set, which was decayed by a factor of 0.1 every 10 epochs. The epochs in the training stage were 20 in total. Considering the impact of batch size on the model’s performance, discussion of the training batch size is given in the next section. The whole training, validation, and testing procedures were conducted with Pytorch (v.1.2.0) on NVIDIA Tesla V100 SXM2 graphical processing units.^27^

### 2.4 Basic Block

**Fig. 2:**
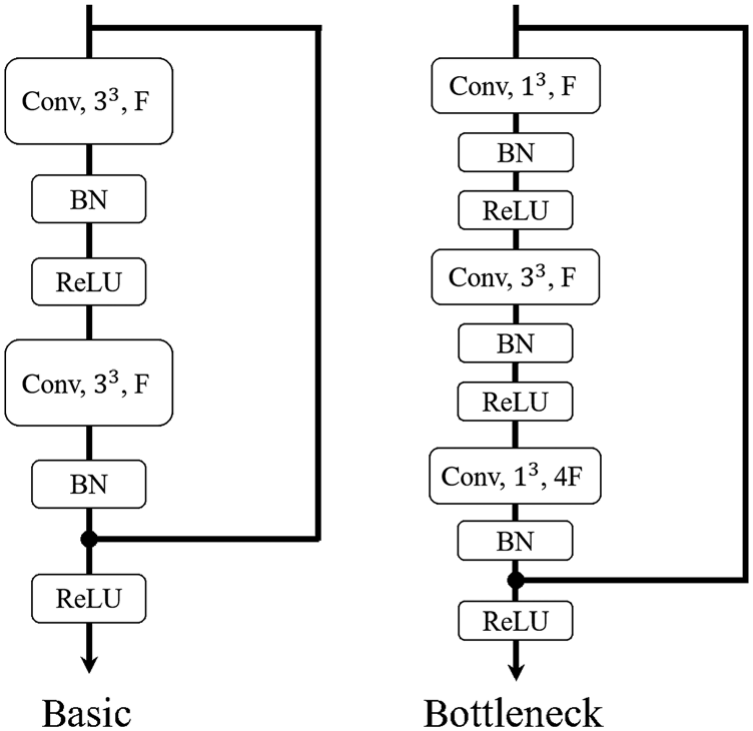
Blocks of architectures. $x^{3}$ denotes the kernel size, and F denotes the number of feature channels.

The basic block of ResNets consists of two convolutional layers (Fig. 2). There are batch normalization and ReLU activation function after each convolutional layer. A shortcut pass connects the top of the block to the layer just before the last ReLU activation function in the block. ResNet-18 and 34 adopt the basic blocks. We use identity connections and zero padding as the shortcuts to the basic blocks to avoid increasing the number of parameters of these relatively shallow networks.^28^

### 2.5 Bottleneck Block

The bottleneck block of ResNets consists of three convolutional layers (Fig. 2). The kernel size of the first and third convolutional layers are 1×1×1, and the second convolutional is 3×3×3. Each convolutional layer is followed by batch normalization and ReLU activation function. The shortcut pass of this block is the same as the basic block. ResNet-50, 101, 152, and 200 all adopt the bottleneck block. Identity connections were used in our model except for those used to increase the dimensions.^28^

### 2.6 (2+1)D Convolutions

**Fig. 3:**
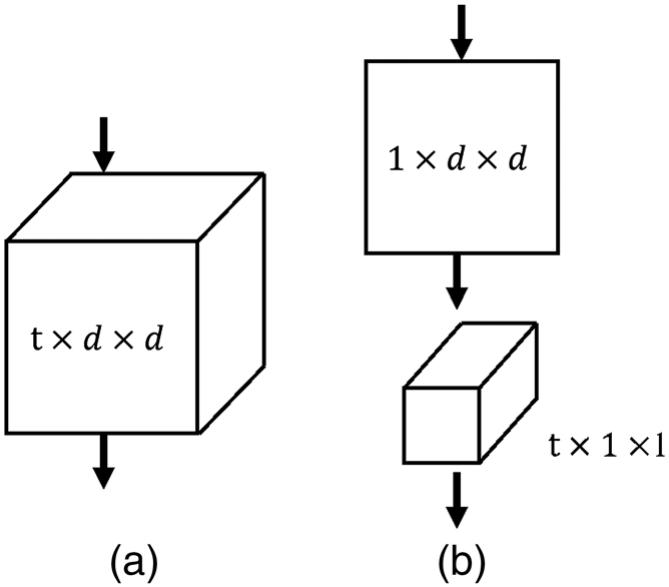
3D versus (2+1)D Convolution. (a) Full 3D convolution and (b) (2+1)D convolution.

R(2+1)D convolution architecture was designed to decompose spatial and temporal modeling into two separate steps by Tran et al.^25^ Whereas the CT slice sequence from the bottom to the top of the same volume block has a similar spatial relation in CT imaging process, the R(2+1)D convolution architecture can be replaced the 3D convolutional filters of size N×t×d×d with a (2+1)D block consisting of 2D convolutional filters of size N×1×d×d and temporal convolutional filters of size N×t×1×1 (Fig. 3).

A nonlinear correction is added between these two operations in the R(2+1)D convolution architecture. Compared with a classical 3D convolutional network using the same number of parameters, this effectively doubles the number of nonlinearities, allowing the model to represent more complex functions. And the decomposition from the R(2+1)D architecture helps to optimize, resulting in lowering both training loss and test loss in practice.

### 2.7 Classification Performance Analysis

The accuracy of a classifier determines the correctness of the predicted value, the precision determines the repeatability of the measurement or the correctness of the predicted value, and the recall or sensitivity indicates how many of the correct results are discovered. The F1-score is used as an overall measure of the model accuracy, combining precision and recall metrics to calculate a balanced average result. First, we calculated the accuracy, recall, precision, and F1-sorce of COVID-19 compared with the two other types. The binary classification formulas for these values are summarized as Eqs. (1) to (4), where TP, TN, FP, and FN are true positive, true negative, false positive, and false negative, respectively, with the positive corresponding to COVID-19 and the negative corresponding to the two other classes. Accuracy and the macroaverage area under the receiver operating characteristic curve (macro-AUROC) were calculated for overall three-way classification. The three-way classification accuracy formula is summarized as Eq. (5), where T and N are all truth and fault, respectively. Bootstrap technology is used to calculate metrics’ average and 95% confidence intervals by nonparametric and unstratified resampling of 1000 times.^29^ Fisher’s exact test is employed to investigate if the improvement in results are significant:

$$\text{Accuracy}=\frac{\mathrm{TP}+\mathrm{TN}}{\mathrm{TP}+\mathrm{FP}+\mathrm{TN}+\mathrm{FN}},$$ (1)

$$\text{Precision}=\frac{\mathrm{TP}}{\mathrm{TP}+\mathrm{FP}},$$ (2)

$$\text{Recall}=\frac{\mathrm{TP}}{\mathrm{TP}+\mathrm{FN}},$$ (3)

$$F1-\text{score}=\frac{2\times \text{Precision}\times \text{Recall}}{\text{Precision}+\text{Recall}},$$ (4)

$${\text{Accuracy}}_{\text{three}-\text{way}}=\frac{T}{T+F}.$$ (5)

### 2.8 Smooth Grad-CAM++ Activity Map Algorithm

After the global average pooling layer of 3D ResNet, the full connection layer is used to calculate the score of each class (NCP, CP, and normal controls), which enables us to utilize the Smooth Grad-CAM++ activity map algorithm to visualize the 3D activation map for the 3D ResNet model. Omeiza et al.^30^ designed the Smooth Grad-CAM++ activity map algorithm as an improved version of class activation mapping (CAM). The Smooth Grad-CAM++ activity map algorithm adds Gaussian noise to original tensor inputs to create new input samples and uses the output of the full connection layer by gradients averaging to generate the weights of the target layer. For each sample from the noise samples, the weighted average of feature maps and weights of the target layer are selected to generate a saliency map. The final saliency map is resized to the original tensor size using the average of saliency maps.

## 3 Results

### 3.1 Complete and Segmented CT Slices

Zhang et al.^21^ employed a diagnostic system based on a lung-lesion segmentation model. The diagnosis took the segmented CT slices as an input generated by segmentation networks using the 3D ResNet-18 network, where the depth and batch size are 64 and 8, respectively. To study the performance difference between the complete and segmented CT slices using the classification network only, 3D ResNet-18 is selected as the baseline model to get metrics from different experiment conditions. Due to the small number of segmented CT slices, we used the same number of complete CT slices, which were split in the same way for training, validation, and test (Table 2).

The accuracy is used to evaluate the overall performance for three-way classification. With the same type of complete CT image, the model’s performance of the more slices group is lower than the less slices group. The possible reason for this is different CP proportions in the more slices group and the less one. The slice number of each class is the same in the less slices group and the segmented CT group. The number of CP slices in the less slices group is 365, accounting for only 0.85% (n=42,861), while the number in the more slices group is 44,880, accounting for 32.70% (n=137,256). The small number of CP makes the classification task simple in the less slices group. The model’s performance is higher even if the model does not distinguish between CP and NCP.

With the same number of slices, the accuracy of complete CT is higher than that of segmented, and the recall is higher (Table 4). The possible reasons include the following: (1) the quality control of segmented CT from CC-CCII dataset is not enough, the segmented image’ boundary is not smooth, and some information is lost. (2) The segmented image dataset is class-unbalanced, and the CP accounts for a smaller proportion in the whole segmented CT dataset. (3) The complete CT image may contain more information that can be learned than segmented ones. Therefore, complete CT slices were used for the rest of this study.

**Table 4:** Accuracy and recall of complete and segmented CT Images. Accuracy is for three-way classification. F1-score, precision, and recall are for binary classification for COVID-19 and the two other classes. The results are represented as average value (the lower bound of 95% confidence interval and the upper bound of 95% confidence interval) generated by bootstrap.

Depth and batch size	Slices	Accuracy (95% CI)	F1-score (95% CI)	Precision (95% CI)	Recall (95% CI)
64 8 (complete)	137,256	0.9297 (0.9269, 0.9324)	0.9237 (0.9201, 0.9273)	0.9409 (0.9366, 0.9453)	0.9075 (0.9022, 0.9129)
64 8 (complete, same number)	42,861	0.9822 (0.9568, 1.0000)	0.9819 (0.9558, 1.0000)	0.9815 (0.9454, 1.0000)	0.9826 (0.9461, 1.0000)
64 8 (segmented)	42,861	0.9255 (0.8750, 0.9761)	0.9214 (0.8660, 0.9767)	0.9594 (0.9036, 1.0000)	0.8875 (0.8017, 0.9733)

### 3.2 Depth and Batch Size

**Fig. 4:**
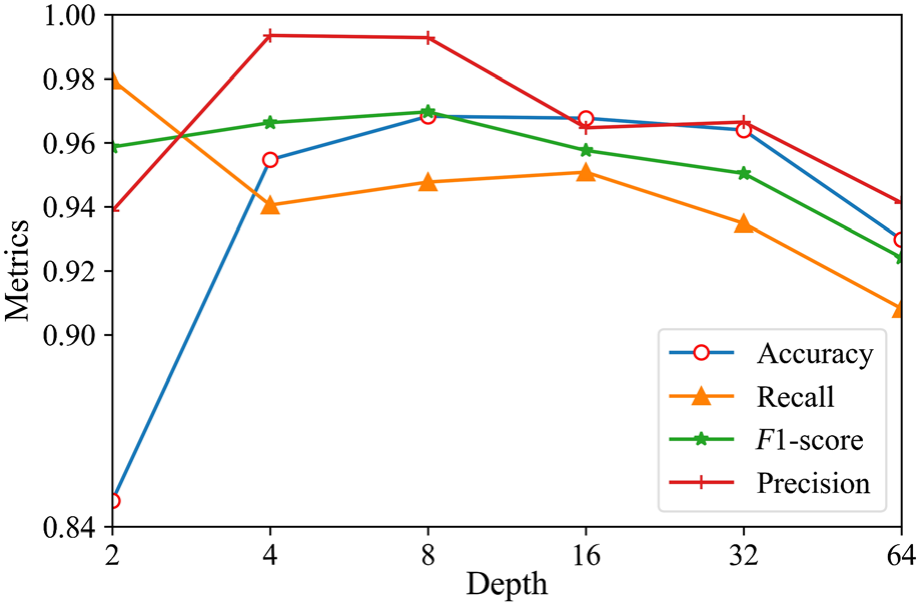
The effect of depths on different metrics.

The depth and batch size have a powerful influence on the model training stage and final accuracy. We first experimented with the three-way classification effect of different depths when the batch size was equal to 8 (Table 5 and Fig. 4). The dataset experiment settings are shown in Table 1.

**Table 5:** Effect of depths on different metrics. Accuracy is for three-way classification. F1-score, precision, and recall are for binary classification for COVID-19 and the two other classes. The results are represented as average value (the lower bound of 95% confidence interval and the upper bound of 95% confidence interval) generated by bootstrap. Bold font indicates the best result. Fisher’s exact test was used to investigate if the improvement in results is significant between the first group and the others. The value of p indicate statistical significance as assessed by two-sided Fisher’s exact tests. “*” means p<0.05, “**” means p<0.01 and “***” means p<0.001.

Depth	Batch size	Accuracy (95% CI)	F1-score (95% CI)	Precision (95% CI)	Recall (95% CI)
2	8	0.8481 (0.8421, 0.8542)	0.9587 (0.9548, 0.9627)	0.9387 (0.9323, 0.9452)	**0.9796** (0.9754, 0.9837)
4	8	0.9547*** (0.9499, 0.9597)	0.9663** (0.9610, 0.9715)	0.9935*** (0.9901, 0.9970)	0.9405*** (0.9310, 0.9500)
8	8	0.9683*** (0.9625, 0.9742)	0.9697** (0.9626, 0.9768)	0.9928*** (0.9877, 0.9978)	0.9477*** (0.9351, 0.9604)
16	8	0.9677*** (0.9556, 0.9798)	0.9576 (0.9402, 0.9751)	0.9647** (0.9431, 0.9863)	0.9508*** (0.9245, 0.9771)
32	8	0.9640*** (0.9550, 0.9730)	0.9504 (0.9373, 0.9634)	0.9665*** (0.9515, 0.9816)	0.9348*** (0.9140, 0.9556)
64	8	0.9298*** (0.9057, 0.9540)	0.9241** (0.8916, 0.9567)	0.9412 (0.9010, 0.9814)	0.9082*** (0.8596, 0.9568)

**Fig. 5:**
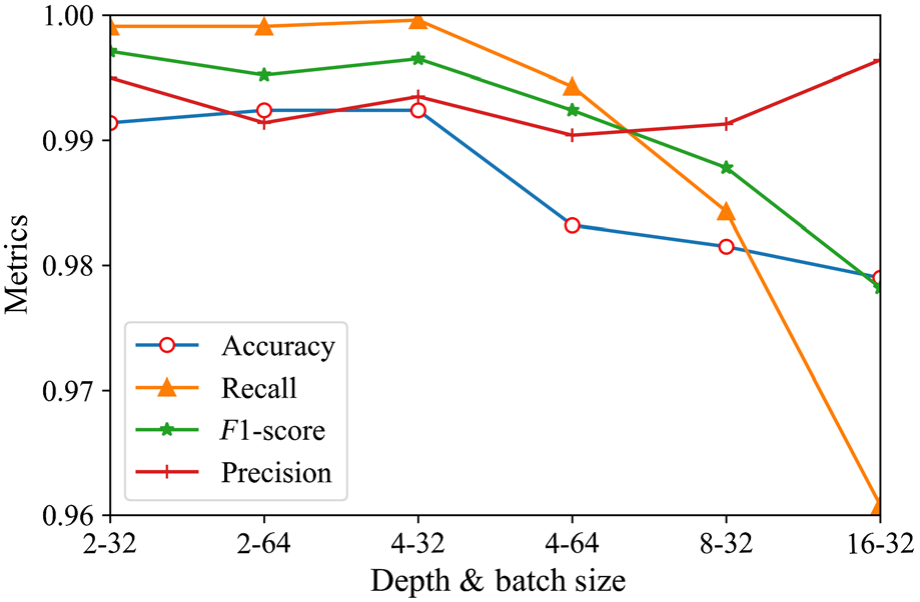
The effect of batch sizes on different metrics.

From Table 5 and Fig. 4, we can conclude that the accuracy and F1-score increase as the depth decreases under the same batch size. We next experimented with the effect of different batch sizes on the performance of the three-classification model (Table 6 and Fig. 5). Due to the limited space on the GPU, we did not use the batch size of 64 for depths 8 and 16.

**Table 6:** Effect of batch sizes on different metrics. Accuracy is for three-way classification. F1-score, precision, and recall are for binary classification for COVID-19 and the two other classes. The results are represented as average value (the lower bound of 95% confidence interval and the upper bound of 95% confidence interval) generated by bootstrap. Bold font indicates the best result. Fisher’s exact test was used to investigate if the improvement in results is significant between the first group and the others. The value of p indicate statistical significance as assessed by two-sided Fisher’s exact tests. “*” means p<0.05, “**” means p<0.01 and “***” means p<0.001.

Depth	Batch size	Accuracy (95% CI)	F1-score (95% CI)	Precision (95% CI)	Recall (95% CI)
2	32	0.9914 (0.9897, 0.9930)	**0.9971** (0.9960, 0.9982)	0.9950 (0.9929, 0.9972)	0.9991 (0.9983, 1.0000)
2	64	0.9924 (0.9910, 0.9939)	0.9952** (0.9939, 0.9966)	0.9914*** (0.9888, 0.9939)	0.9991 (0.9983, 0.9999)
4	32	**0.9924** (0.9902, 0.9945)	0.9965 (0.9948, 0.9982)	0.9935 (0.9903, 0.9968)	**0.9996** (0.9987, 1.0000)
4	64	0.9832*** (0.9802, 0.9862)	0.9924*** (0.9899, 0.9949)	0.9904*** (0.9865, 0.9944)	0.9943*** (0.9913, 0.9974)
8	32	0.9815*** (0.9751, 0.9880)	0.9878*** (0.9815, 0.9941)	0.9913 (0.9839, 0.9986)	0.9843*** (0.9741, 0.9946)
16	32	0.9790*** (0.9743, 0.9838)	0.9782*** (0.9722, 0.9843)	**0.9964** (0.9928, 1.0000)	0.9608*** (0.9497, 0.9718)

**Table 7:** Comparison of classification results using different models. Accuracy is for three-way classification. F1-score, precision, and recall are for binary classification for COVID-19 and the two other classes. The results are represented as average value (the lower bound of 95% confidence interval and the upper bound of 95% confidence interval) generated by bootstrap. Bold font indicates the best model group. Fisher’s exact test was used to investigate if the improvement in results is significant between the first group and the others. The value of p indicate statistical significance as assessed by two-sided Fisher’s exact tests. “*” means p<0.05, “**” means p<0.01 and “***” means p<0.001.

Model	Accuracy (95% CI)	F1-score (95% CI)	Precision (95% CI)	Recall (95% CI)
**ResNet-18**	0.9924 (0.9902, 0.9945)	0.9965 (0.9948, 0.9982)	0.9935 (0.9903, 0.9968)	0.9996 (0.9987, 1.0000)
ResNet-18(2+1)D	0.9885* (0.9860, 0.9910)	0.9957 (0.9938, 0.9976)	0.9935 (0.9903, 0.9968)	0.9978** (0.9959, 0.9997)
ResNet-34	0.9800*** (0.9767, 0.9834)	0.9816*** (0.9776, 0.9856)	0.9920 (0.9884, 0.9956)	0.9714*** (0.9645, 0.9783)
ResNet-34(2+1)D	0.9719*** (0.9679, 0.9758)	0.9769*** (0.9725, 0.9814)	0.9911 (0.9871, 0.9951)	0.9632*** (0.9555, 0.9709)
ResNet-50	0.9801*** (0.9768, 0.9834)	0.9831*** (0.9793, 0.9870)	0.9942 (0.9912, 0.9973)	0.9723*** (0.9655, 0.9791)

From Table 6 and Fig. 5, we conclude that the accuracy and F1-score are significantly improved as the batch size is increased to 32. In particular, when the depth is 2 and 4, the accuracy and F1-score reached 0.99, but increasing the batch size to 64 does not further improve the model performance and the accuracy and recall rate even decrease.

### 3.3 Different Models

According to the conclusion in Table 6, we use optimal parameters of 4 for depth and 32 for batch size to train different models, including 3D ResNet-34, 3D ResNet-50, (2+1)D ResNet-18, and (2+1)D ResNet-34. The 3D ResNet-18 model used the best performance group (4 for depth and 32 for batch size) and results from Table 6. Table 7 shows the results of different models.

As the number of 3D ResNet layers deepened to 34, the network appeared to overfit and the accuracy and recall dropped slightly. The performances of 3D ResNet-34 and 3D ResNet-50 were relatively close. After replacing the 3D convolution with (2+1)D, the accuracy decreased.

**Fig. 6:**
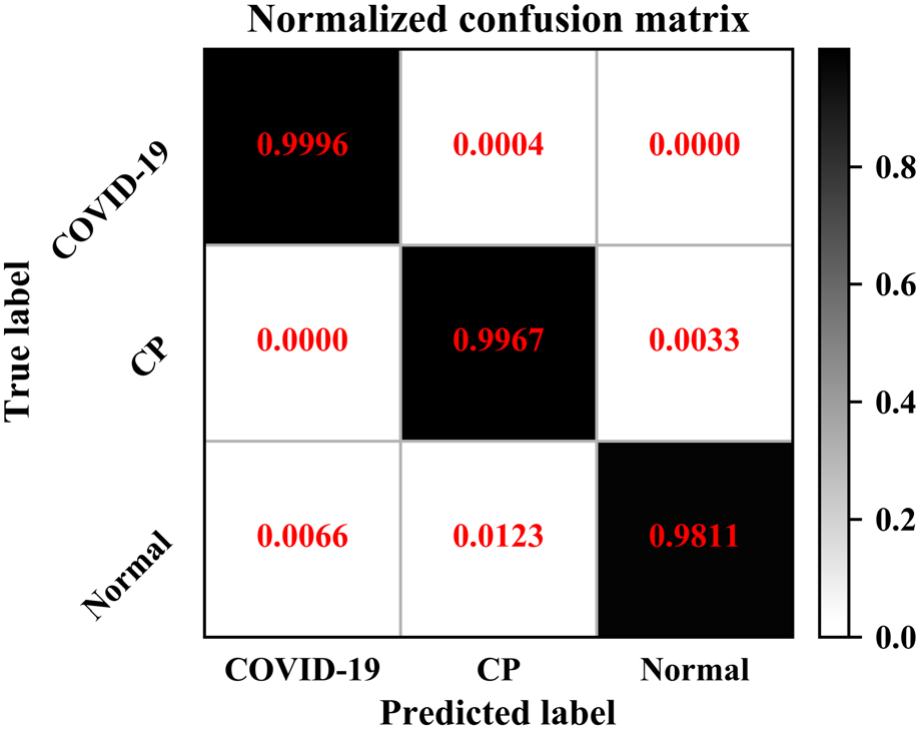
Normalized confusion matrix of depth 4 and batch size 32. The model used is 3D ResNet-18.

Our optimal model, which is from the 3D ResNet-18 network was able to discriminate COVID-19 from the two other classes (other CP and normal controls) with 99.76% accuracy, 99.96% recall, 99.35% precision, and 99.65% F1-score (Fig. 6). The overall performance for three-way classification obtained 99.24% accuracy and macro-AUROC of 0.9998.

### 3.4 Visualization

Although our model achieved high performance in CC-CCII dataset, it is still a black box model. As a discriminative model, the CNN only receives input and gives high accuracy output but cannot give the basis of prediction. The auxiliary information for doctors’ diagnosis is very limited because it cannot provide a decision-making basis. We use the Smooth Grad-CAM++ activity map algorithm to inspect the model’s inner mechanism. We apply the Smooth Grad-CAM++ algorithm on a single slice from the volume of each class via the 3D ResNet model (depth is 4, batch size is 32) with the target layer at the last convolution layer before the global average pooling layer. Regions that appear purple and brighter have a larger impact on the model’s decision to classify a slice to its own class.

**Fig. 7:**
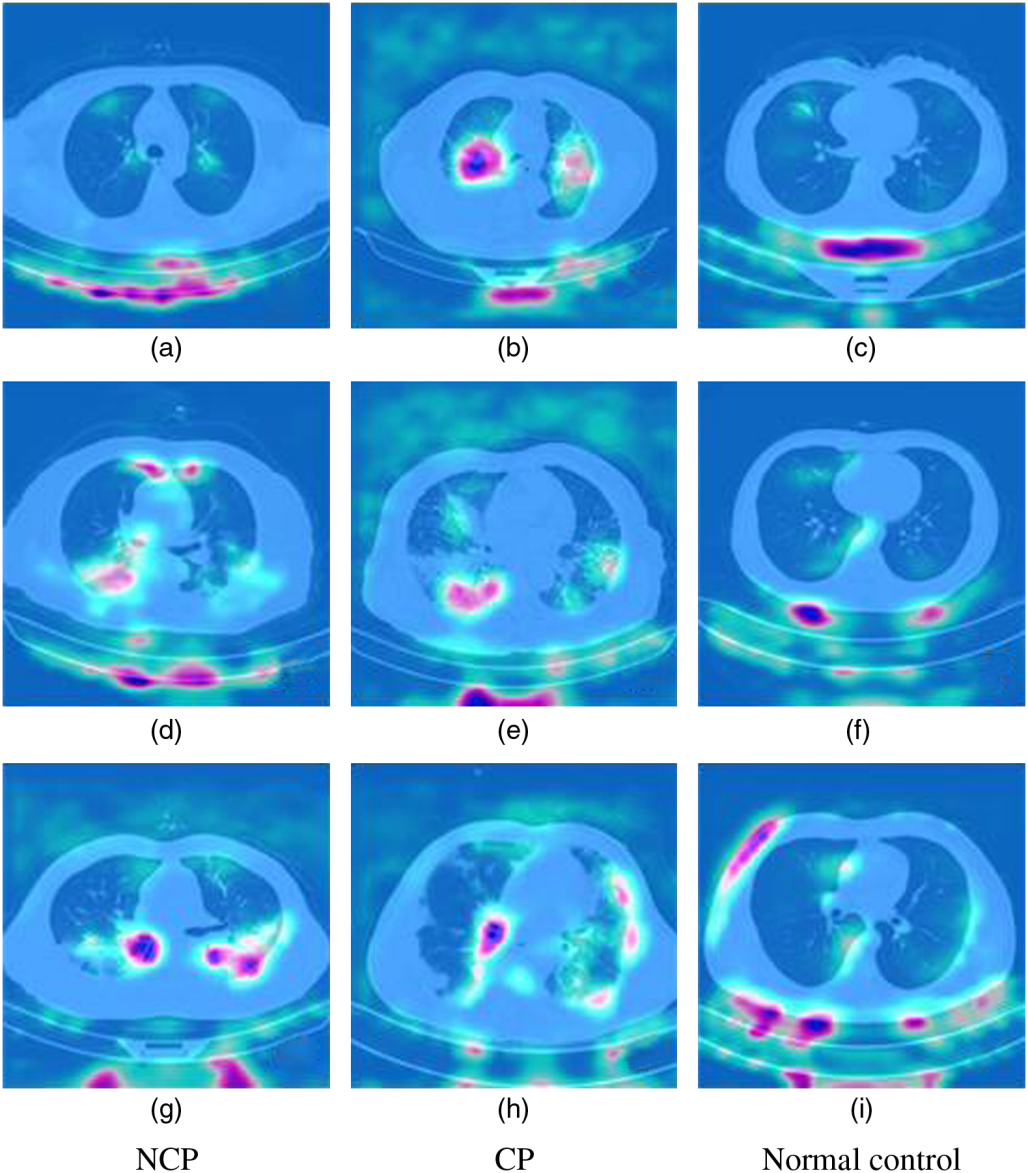
Saliency maps of the Smooth Grad-CAM++ algorithm. (a), (d), (g) The first column contains slices with NCP, (b), (e), (h) the second column contains slices with CP, and (c), (f), (i) the third column contains slices in the normal control group.

The model focuses on some lung edges and messy positions in the normal control case (C, F, and I). Compared with the normal control case, the GGO, and pulmonary consolidation (CL) area on the slice can provide significant information for the doctor to diagnose COVID-19 and CP. From Fig. 7, we can see that the model pays more attention to the GGO and CL area accurately no matter whether it is dispersed [Figs. 7(a), (d), (e), (h)] or gathered [Figs. 7(b) and (g)].

The clinical manifestations and radiological findings of NCP and CP are similar. It is difficult to identify them by a CT scan only. Figure 7 shows that COVID-19 is more related to GGO located in the subpleural area, and CP is more related to the block of CL. The lesion area on the CT image is some small GGO areas along the bronchovascular bundle or located in the subpleural area in the early stage of COVID-19 (A). However, considering the sample capacity of the CC-CCII dataset, visualization has its limitations, and the specific imaging features of COVID-19 and CP need further study.

**Fig. 8:**
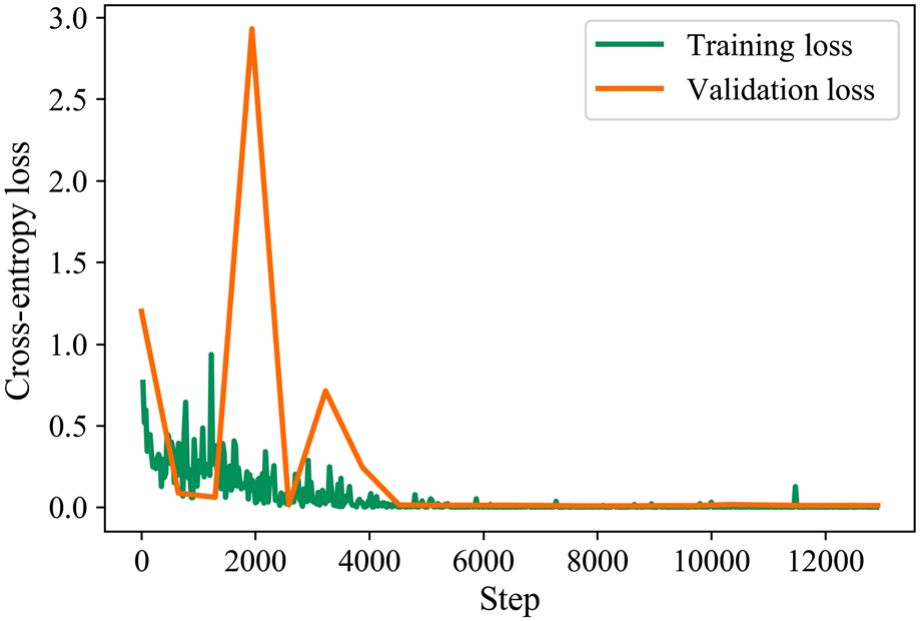
Training and validation losses in the training stage.

**Fig. 9:**
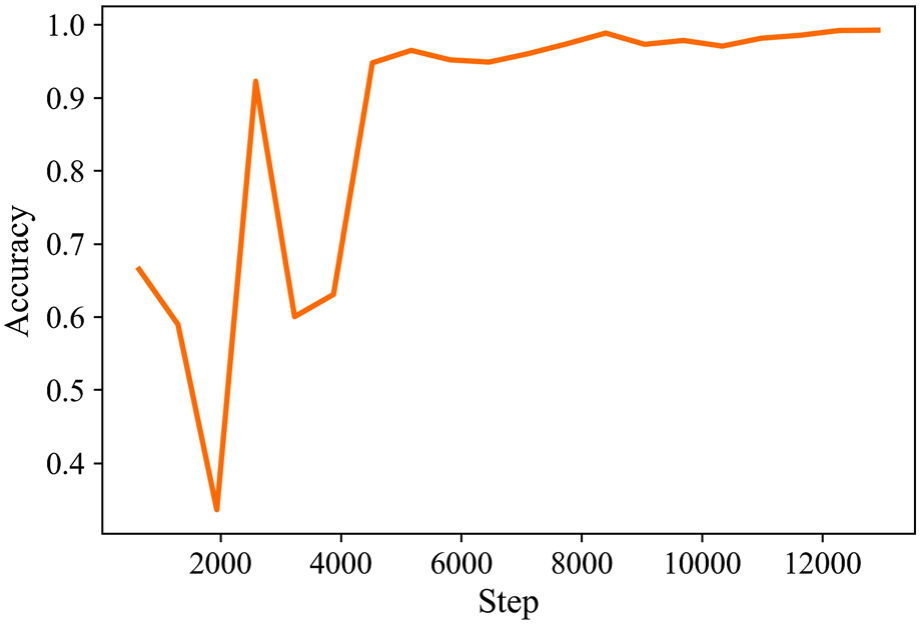
Accuracy curve of training and validation sets in the training stage.

Figures 8 and 9 describe the training loss, validation loss, and accuracy curves of training and validation of the best performance model during the training stage in 20 epochs.

## 4 Discussion

For COVID-19, getting a diagnosis as soon as possible is essential. As a powerful tool, CT provides chest scans in a short time. In this study, we presented a deep-learning method for automatic diagnosis of COVID-19 from chest CT images to assist clinicians and radiologists in combating this pandemic.

According to the depth and batch size parameters’ setting of Zhang et al.,^21^ the classification network is used for three classifications, instead of the combination of segmentation network and machine learning models. The results show that model’s performance of using the complete CT is better than that of the segmentation CT, which shows that the complete CT can provide more information than the segmented CT in the end-to-end classification task of COVID-19 and other kinds of pneumonia. Due to the lack of image annotation, we cannot compare the end-to-end classification network model with the machine learning model based on lung-lesion features. Next, we plan to extract the lesion region information based on segmentation methods and then make the corresponding comparison.

To find out whether the depth and batch size settings are suitable for the 3D classification network, we carried out experiments to explore the optimal parameters of depth and batch size. Combining different depth and batch sizes, we obtained a series of results and found that the model’s performance is the highest when the depth is 4 and the batch size is 32. The series of experiments did not change the basic parameter settings of the classic 3D ResNet model structure; they only modified the dimension of the data batch input. The results are convincing only when the baseline model is discussed. For a model with a different structure, we intend to study further.

Using the model with the highest performance for visualization, we found that the model can focus on the GGO on the edge of the lung boundary. However, there are some messy locations in all three class cases, and we speculate that the noise area is used to determine the position of a slice in volume.

## 5 Conclusions

In this work, we designed a deep-learning method using CT images to classify COVID-19, CP, and normal controls. We employed a variety of 3D ResNet models and finally determined the best model as 3D ResNet-18. Experimental results show that 3D ResNet-18 is the best model for distinguishing COVID-19 from CP and normal controls at the CC-CCII dataset. We proposed a preprocessing method that was to superimpose CT slices into volumes of different depths. We raised the issue of the impact of depth on classification performance and proved that depth 4 had the largest improvement in model performance instead of 64. A total of 110,420 complete CT images (80.4%) were employed to train and validate our model, and the remaining 26,836 CT images (19.6%) were used as the test set. Our model has a high performance, achieving recall of 99.96%, precision of 99.35%, F1-sorce of 99.65%, three-way classification accuracy of 99.24%, and macro-AUROC of 0.9986. We believe that our model’s high performance can be attributed to a large, high-quality dataset that we employed and different depths used to train 3D models. Our deep-learning model can alleviate the significant need for diagnostic expertise when the health system is overburdened in pandemic situations or remote areas. Currently, our model is designed to help radiologists and clinicians as an effective first-time screening tool as this can reduce patient waiting time and shorten diagnostic workflow time, thereby lessening the overall workload of radiologists and enabling them to respond quickly and effectively in emergency situations.
